# Roger W. Sperry (1913–1994)

**DOI:** 10.1007/s00415-022-11232-6

**Published:** 2022-07-22

**Authors:** Stefano Sandrone

**Affiliations:** Department of Brain Sciences, Imperial College London, London, UK

**Fig. 1:**
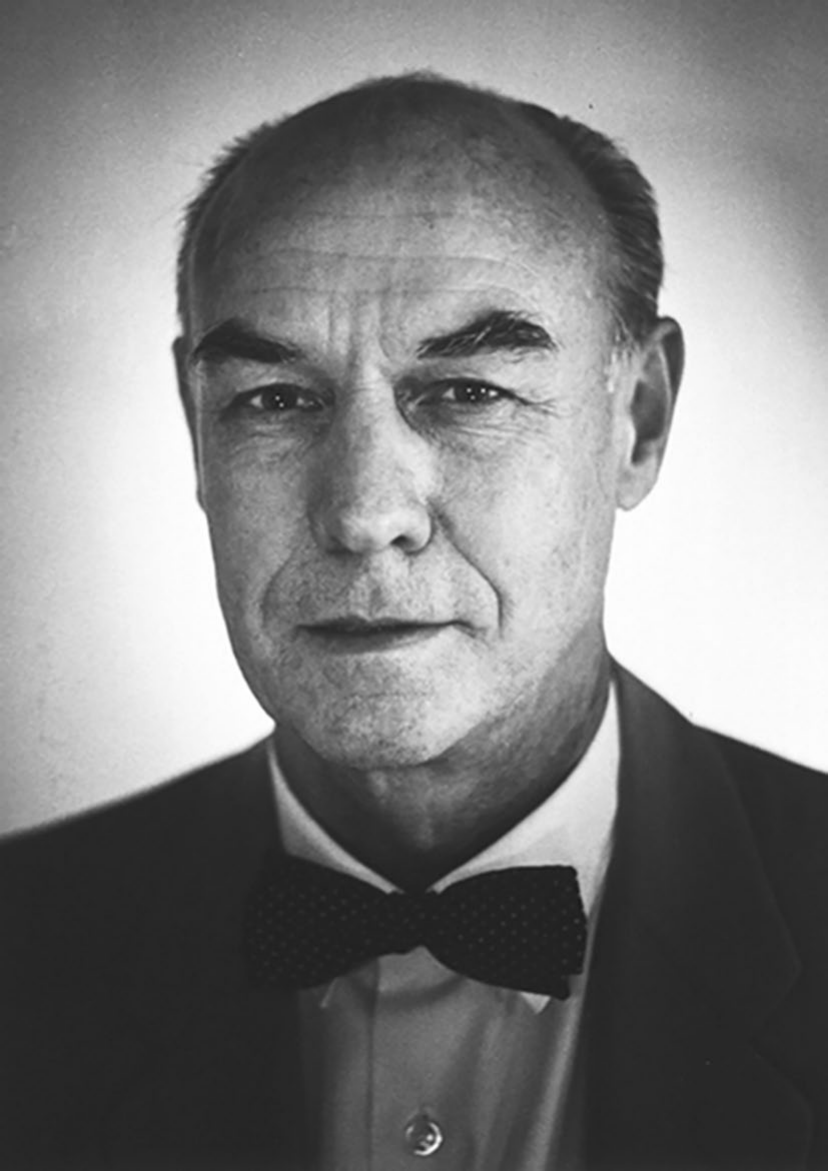
Portrait of Roger Sperry. Credits: National Library of Medicine, Digital Collections. Courtesy of the National Library of Medicine

Roger Wolcott Sperry made titanic contributions to neurobiology [1]. He did so by challenging some of the existing scientific dogmas and making discoveries that revolutionised the fields of neural development and hemispheric specialisation [2], Fig. 1. Sperry was born on the 20th of August 1913 in Hartford, Connecticut, and spent his early years on a nearby farm with his family [3]. His father, Francis Bushnell, worked in the banking business, but died when Roger was 11 years old [4]. After this, Roger’s mother, Florence Kraemer Sperry, relocated with the family to West Hartford and started to work as an assistant to the principal in a high school to look after Roger and his 1-year younger brother, Russel Loomis [4]. At Hall High School, Sperry showed his academic talent and excelled in several sports—he even set a state record in the javelin throw [2]. He won an Amos C. Miller Scholarship to join Oberlin College, where a Sperry Neuroscience Wing in the Kettering Hall building was inaugurated in 1990. At Oberlin College, he was the captain of the basketball team and continued to excel in sports [3]. Over the years, he matured a curiosity in palaeontology, and, in his free time, he was a painter, sculptor and ceramist.

But at the core of his interests there was psychology, a passion reportedly spurred by an introductory module on psychology given by R. H. Stetson, a leading authority on the physiology of speech [2] who studied at Harvard with William James [5]. During his psychology course as a freshman, Sperry wrote the following questions on the first page of his class notes: ‘Where does behaviour come from? What is the purpose of consciousness?’ [3, 5]. In many ways, these questions shaped his research activities for the coming years. Sperry completed his undergraduate degree in English (1935) before obtaining his master’s degree in psychology (1937). He then completed his PhD in zoology at the University of Chicago in 1941 with neuroembryologist Paul A. Weiss, and, between 1942 and 1945, he also performed ‘military service on the Medical Research Project on Nerve Injuries’ [6]. His experiments continued with Karl Lashley at Harvard and the Yerkes Laboratories in Orange Park, Florida [4, 6] until 1946, when he joined the University of Chicago as an assistant professor [4].

In 1949, he married Norma Gay Deupree; they had a son, Glenn Michael (Tad), and a daughter, Janeth Hope [4]. In 1952, Sperry was appointed as the Section Chief of Neurological Diseases and Blindness at the National Institutes of Health and then associate professor in Chicago. Although he planned to move to Bethesda, in 1954 he accepted an offer from the California Institute of Technology, where he became the inaugural Hixson Professor of Psychobiology.

Throughout his career, by adopting an experimental approach permeated by simplicity and lucidity [1], he challenged the ideas and theories of his doctoral and postdoctoral advisors, leading scientists in their fields [3]. At that time, it was widely believed that the developing nervous system was ‘a random, diffuse, unstructured, and essentially equipotential transmission network, a blank slate to be (…) shaped into a functionally adaptive communication system by use, experience, practice and learning’ [7]. Sperry performed surgical resections on fish, frogs, salamanders and rats to demonstrate, contrary to Paul Weiss’s theory, that nerves were guided to re-establish the same connections, whether it was the retina-brain connection or a foot innervation. Even interchanging the tendinous insertions of extensor and flexor muscles (and the nerves supplying them) did not lead to a re-learning [1]. With his chemoaffinity hypothesis, he echoed Cajal’s theoretical concepts of neurotropism and chemical selectivity [7]. These findings [8] make him a pioneer of the studies on axonal guidance. But it is not for these works that he won the Nobel Prize.

The corpus callosum, the largest commissural tract of the brain, connects the two hemispheres, but its functions were not known in the 1950s. Sperry and his PhD student Ronald Myers assessed visual deficits following the resection of the corpus callosum in cats [7]. This was the first chapter of a new research line on ‘split brains’, with studies also conducted in humans, to explore neurocognitive callosal functions in subjects surgically treated to limit the diffusion of epilepsy between the hemispheres [5]. Pioneering split-brain experiments, performed with Joseph Bogen and Michael Gazzaniga, showed that the right cerebral hemisphere has language abilities too and can perform visuospatial tasks better than the left one [1, 9, 10]. Other elegant works shed light on the duality of consciousness emerging after severing the corpus callosum, which stopped the information transfer between the two hemispheres; Sperry and co-workers opened up a new field on mind and consciousness at the crossroads between neuropsychology and philosophy, neuroscience and metaphysics [7]. He rejected Cartesian dualism, supported a mentalism theory and believed that consciousness was a ‘supervenient result of neural activity’ [7], emerging independently from the chemistry and biophysics of neurons [2].

Sperry won the Nobel Prize in 1981 for his breakthrough discoveries on functional specialisation of the hemispheres; the other half of the prize was jointly given to David Hubel and Torsten Wiesel for their studies on visual information processing. Among the many awards and honours received, Sperry was elected to the National Academy of Sciences in 1960, was elected Foreign Member of the Royal Society in 1976 and became a member of the Pontifical Academy of Sciences in 1978. In the following year, he was awarded the Albert Lasker Medical Research Award and the Wolf Prize in Medicine, and in 1989 he won the National Medal of Science.

After retiring, while suffering from a progressive neuromuscular degenerative disease, he continued working on consciousness, theories of mind and the mind-brain problem. He was described as a tenacious researcher [5] and a shy [2], taciturn person [6] who enjoyed boating, fishing and collecting fossils [4] and was in love with the beauty of remote places [3]. Sperry died after a heart attack on the 17th of April 1994 in Pasadena, California.
